# Comparative Leaf Anatomy of Balkan Representatives of *Gentiana* L. Sect. *Ciminalis* (Adans.) Dum. (Gentianaceae): Implications for Species Delimitation

**DOI:** 10.3390/plants14192977

**Published:** 2025-09-25

**Authors:** Žarko Mladenović, Nevena Kuzmanović, Dmitar Lakušić, Danilo Stojanović

**Affiliations:** 1 Department of Botany, Faculty of Pharmacy, University of Belgrade, Vojvode Stepe 450, 11221 Belgrade, Serbia; danilo.stojanovic@pharmacy.bg.ac.rs; 2 Institute of Botany and Botanical Garden, Faculty of Biology, University of Belgrade, Studentski trg 16, 11158 Belgrade, Serbia; nkuzmanovic@bio.bg.ac.rs (N.K.); dlakusic@bio.bg.ac.rs (D.L.)

**Keywords:** anatomy, Balkan Peninsula, differentiation, *Gentiana* sect. *Ciminalis*

## Abstract

The present study investigates the leaf anatomical traits of representatives of *Gentiana* section *Ciminalis* in the Balkan Peninsula, focusing on the ecologically and geographically vicariant species *Gentiana acaulis*, *G. clusii*, and *G. dinarica*. These species are distributed across a variety of mountainous habitats, including calcareous and siliceous rocky grounds, and exhibit pronounced morphological similarities that have led to misidentifications in the past. In order to address the challenges in species delimitation, a comparative analysis of leaf anatomical traits was performed on cross-sections of ten rosette leaves from each population. Statistical data analyses were conducted on 18 morphometric traits. A range of statistical techniques were used to assess variability and identify important discriminating traits, including descriptive statistics, principal component analysis, and discriminant analysis. The results indicate that the species can be distinguished based on leaf anatomy, particularly mesophyll thickness and number of cells that contain calcium oxalate crystals. The leaf of *G. acaulis* has a smaller mesophyll thickness (mean value: 164.31 μm), *G. dinarica* a larger mesophyll thickness (mean value: 365.85 μm), while *G. clusii* lies between these two (mean value: 305.35 μm). Crystal-containing cells are most abundant in *G. clusii*, where they are distributed throughout the entire leaf mesophyll; followed by *G. dinarica*, where the distribution of these cells are mainly in the upper half of the leaf; while they are sparse or absent in *G. acaulis*. These results suggest that leaf anatomy is a valuable diagnostic tool for distinguishing taxa within the section *Ciminalis* of the genus *Gentiana*.

## 1. Introduction

The genus *Gentiana* L. comprises over 360 species classified into 15 sections. It is the most diverse genus among the 99 genera from the family Gentianaceae [1,2,3]. The genus is subcosmopolitan, with species predominantly distributed in the mountainous regions of Eurasia. A smaller part of its range (with a small number of species) extends to North and South America, Northwest Africa, and Eastern Australia [4]. The centre of *Gentiana* diversity is located in the Tibeto-Himalayan region, where more than 250 species have been documented [5].

Although *Gentiana* is a morphologically well-defined group that appears to be monophyletic [6], the synonymizations and subsequent reclassifications of various species over time have emphasised the intricate taxonomy and complexity of this genus [4,7].

This is particularly evident in the section *Ciminalis* (Adans.) Dumort., which includes seven ecologically and geographically vicariant and closely related species: *Gentiana acaulis* L., *Gentiana alpina* Vill., *Gentiana angustifolia* Vill., *Gentiana clusii* E. M. Perrier & Songeon, *Gentiana dinarica* Beck, *Gentiana ligustica* R. Vilm. & Chopinet, and *Gentiana occidentalis* Jakow. These species are distributed across the mountainous regions of Southern and Central Europe, with the Alps representing the diversity centre of the section [3,4,7,8,9]. Within this group, two calcifuge species, *G. acaulis* and *G. alpina*, exhibit an ecologically vicariant distribution pattern in relation to the five calcicole species. Of these five calcicole species, *G. angustifolia*, *G. dinarica*, *G. ligustica*, and *G. occidentalis* are geographically vicariant, while *G. clusii* exhibits a wide distribution that extends from the Pyrenees to the Carpathians, with partial overlap with the distribution areas of the other calcicole species [7].

**Figure 1:**
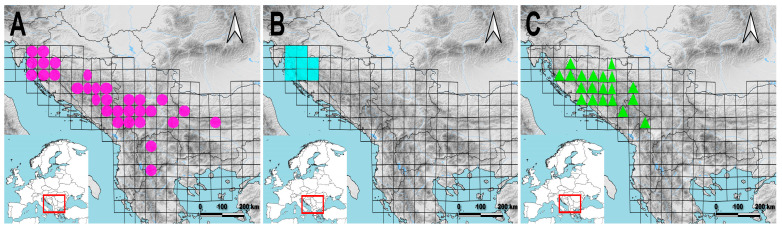
Distribution of *Gentiana acaulis* (**A**), *Gentiana clusii* (**B**), and *Gentiana dinarica* (**C**) on the Balkan Peninsula given in MGRS, grid cells 50 × 50. Based on [4] and field work of the authors.

The Balkan Peninsula harbours three species of this section: *G. acaulis*, *G. clusii*, and *G. dinarica* [4] (Figure 1). *Gentiana clusii* thrives on calcareous rocky grounds and grasslands in the upper montane and subalpine zones. It is vicarious with the related species *G. acaulis*, which favours siliceous shallow stony grounds or deep soils from which the lime has been removed by washing. In contrast to the two previous species in the section, which predominantly inhabit mountainous, rocky grounds on limestone (*G. clusii*), or silicate (*G. acaulis*), *G. dinarica* is typically found on steep rocky outcrops of stony calcareous or dolomite and generally inhabits more rocky environments compared to *G. clusii* and *G. acaulis* [4]. These three species exhibit pronounced morphological similarities, which have led to their misidentifications in the past. The most reliable distinguishing features are the corolla coloration, the shape and size of the calyx lobes, and the shape of the leaves [8,10]. The recognition of *G. acaulis*, *G. clusii*, and *G. dinarica* as distinct species by various authors and checklists is well-documented [2,3,4,7,8,9,11]. However, in other checklists [12,13] *Gentiana dinarica* is treated as a subspecies of *G. acaulis* based on the new combination proposed by Barina et al. [14]—*G. acaulis* ssp. *dinarica* (Beck) Barina.

Considering the available literature data on the three species of *Gentiana* section *Ciminalis*, it is important to note that their chorological and ecological relationships in the western–central part of the Balkan Peninsula are not fully clarified. The observed morphological similarities among the studied species emphasise the need for further investigation to resolve challenges related to their delimitation. Previous studies of leaf anatomy have exclusively focused on individual species from this section [15,16]. However, comparative studies of leaf anatomical features of all representatives are lacking. Therefore, the main aim of our study was to examine the anatomical features of the leaves of all Balkan representatives of *Gentiana* section *Ciminalis*, and to identify those that could facilitate species delimitation.

## 2. Results

### 2.1. General Characteristics of the Leaf Anatomy

**Figure 2:**
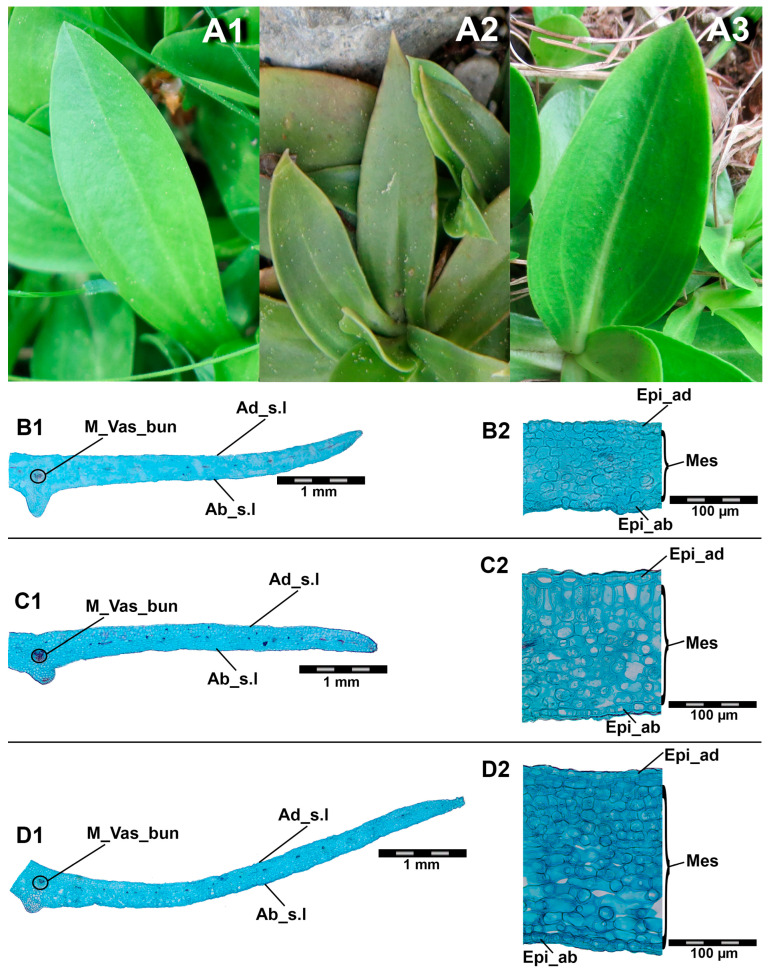
(**A1**) Leaf of *G. acaulis*; (**A2**) leaf of *G. clusii*; (**A3**) leaf of *G. dinarica*. (**B1**) Entire cross-section of the leaf of *G. acaulis* (magnification 2×); (**B2**) detail of the cross-section of the leaf of *G. acaulis* (magnification 20×); (**C1**) entire cross-section of the leaf of *G. clusii* (magnification 2×); (**C2**) detail of the cross-section of the leaf of *G.clusii* (magnification 20×); (**D1**) entire cross-section of the leaf of *G. dinarica* (magnification 2×); (**D2**) detail of the cross-section of the leaf of *G. dinarica* (magnification 20×). M_Vas_bun—main vascular bundle, Ad_s.l—adaxial side of the leaf, Ab_s.l—abaxial side of the leaf, Epi_ad—epidermis on the adaxial side of the leaf, Epi_ab—epidermis on the abaxial side of the leaf, Mes–mesophyll.

Rosette leaves of all three species are sessile (Figure 2(A1–A3)). The shape of the leaves varies between the species: in *G. acaulis* they are lanceolate, elliptical or, less frequently, obovate (Figure 2(A1)); in *G. clusii* they are elliptical to oblong-lanceolate (Figure 2(A2)); while in *G. dinarica* they are broadly elliptical (Figure 2(A3)). The laminar organisation of the leaf of the examined species is generally characterised by a dorsiventral arrangement with a partial differentiation of mesophyll to palisade and spongy parenchyma (Figure 2(B1–D2)). In *G. acaulis*, the entire mesophyll is composed of isodiametric chlorenchyma cells (Figure 2(B1,B2)). While the cells are not arranged in the standard palisade and spongy tissue patterns, the presence of intercellular spaces parallels the organisation of these tissue types as the first 2–3 layers of these cells beneath the upper epidermis lack intercellular spaces, while the following 4–5 layers contain small intercellular spaces. The mesophyll of *G. dinarica* (Figure 2(D1,D2)) is also composed predominantly of isodiametric cells. As in the previous species, the first 3–4 cell layers below the upper epidermis lack intercellular spaces; however, in contrast to *G. acaulis*, the subsequent 4–8 cell layers resemble typical spongy parenchyma with large intercellular spaces. The separation of mesophyll to palisade and spongy parenchyma is most clearly observed in *G. clusii* (Figure 2(C1,C2)). The first 3–4 layers below the upper epidermis have no intercellular spaces, and 1–2 of these layers consist of cells that are longitudinally elongated and perpendicular to the cells of the upper epidermis. The next 4–6 layers of isodiametric cells have large intercellular spaces that resemble the typical spongy parenchyma.

**Figure 3:**
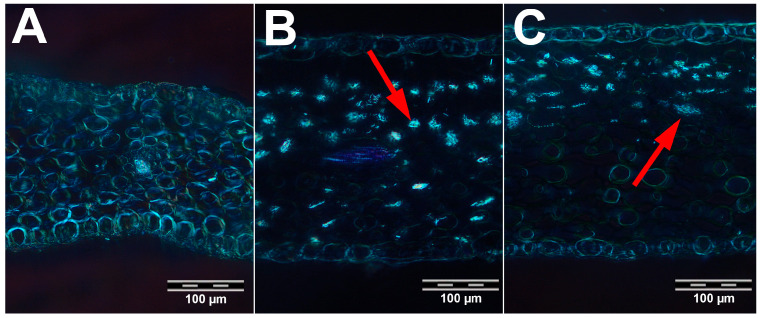
Cells with calcium oxalate crystals in three species of *Gentiana* section *Ciminalis*. (**A**) *Gentiana acaulis*. (**B**) *G. clusii*. (**C**) *G. dinarica*. Red arrows indicate crystal-containing cells. (Magnification 20×).

The surface area of the half leaf cross-section (multiplied by two in the statistical analysis) (A_L) ranges from 0.73 to 7.28 mm^2^. The thickness of the leaf in the area of the central nerve (T_LC) varies between 343.69 and 1334.38 µm, and the thickness between the central nerve and the leaf margin (T_LH) varies between 141.76 and 572.91 µm (Table 1). The epidermis on the adaxial and abaxial side of the leaf is single-layered and approximately the same thickness. The thickness of the epidermis on the adaxial side (T_Epi_ad) ranges from 13.99 to 36.56 µm, while the thickness on the abaxial side (T_Epi_ab) ranges from 13.42 to 32.16 µm (Table 1). The thickness of the epidermis at the leaf margins (T_Epi_ma) is greater than on the adaxial and abaxial sides and varies between 17.82 and 102.78 µm (Table 1). There is a thin layer of cuticle on both sides of the epidermis, with the thickness ranging from 2.57 to 9.62 µm on the adaxial side (T_Cut_ad) and from 2.42 to 8.45 µm on the abaxial side (T_Cut_ab) (Table 1). A greater thickness of the cuticle at the leaf margins (T_Cut_ma) was observed, with measurements ranging from 3.92 to 17.59 µm (Table 1). While the mesophyll is not differentiated in the populations belonging to *G. acaulis*, palisade and spongy parenchyma can be distinguished in most populations belonging to *G. clusii* and *G. dinarica*. The thickness of the mesophyll (T_Mes) ranges from 102.55 to 504.57 µm (Table 1). The vascular bundles in all examined populations of the three species are of collateral type. The perimeter of the main vascular bundle (P_CN) ranges from 39.36 to 112.74 µm (Table 1). The mesophyll may contain cells with calcium oxalate crystals (Figure 3). The number of these cells on the 1 mm-long portion of the leaf cross-section (No_CC) varies from 0 to 210 (Table 1). Descriptive statistics of all character states are shown in Table 1.

### 2.2. General Characteristics of the Leaf Epidermis

**Figure 4:**
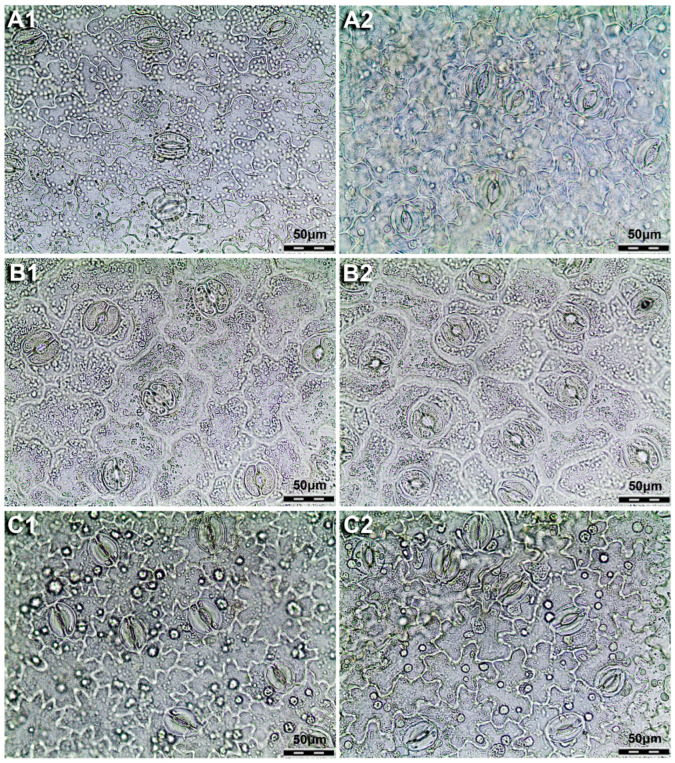
Epidermis and stomata of the three examined species of *Gentiana* section *Ciminalis*. (**1**) Epidermis of adaxial side of the leaf; (**2**) epidermis of abaxial side of the leaf; (**A**) *G. acaulis*; (**B**) *G. clusii*; (**C**) *G. dinarica*. (Magnification 40×).

Leaves are amphistomatous in all three species. Type of stomata within all three species is anomocytic (Figure 4). Number of stomata on both sides of the leaf is counted on a surface area of 0.324 mm^2^. Number of stomata on the adaxial side of the leaf (No_Stom_ad) ranges from 7 to 45, while on the abaxial side their number (No_Stom_ab) varies from 16 to 58. Length of the stoma on the adaxial side of the leaf (L_Stom_ad) ranges from 28.5 to 43.1 µm and on the abaxial side its length (L_Stom_ab) varies from 31.3 to 42.69 µm. Width of the stoma on the adaxial side of the leaf (W_Stom_ad) varies between 23.41 and 36.9 µm, while on the abaxial side (W_Stom_ab) it ranges from 23.3 to 35.36 µm (Table 1, Figure 4).

**Figure 5:**
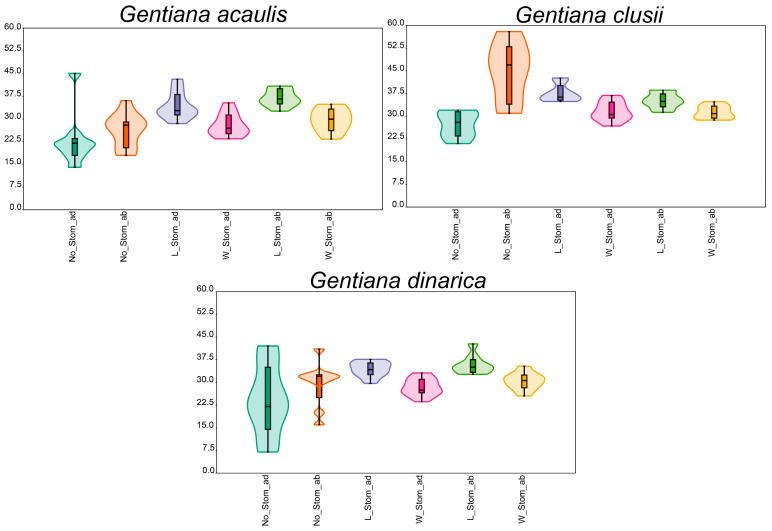
Box and violin plots of quantitative characters of leaf epidermis. No_Stom_ad—number of stomata on the adaxial side of the leaf; No_Stom_ab—number of stomata on the abaxial side of the leaf; L_Stom_ad—length of the stomata on the adaxial side of the leaf; L_Stom_ab—length of the stomata on the abaxial side of the leaf; W_Stom_ad—width of the stomata on the adaxial side of the leaf; W_Stom_ab—width of the stomata on the abaxial side of the leaf.

Out of all analysed characters, it was found that the number of stomata, especially on the abaxial side of the leaf (No_Stom_ab), was the only variable that allowed for the clear differentiation of the species (Table 2, Figure 5). The species *G. clusii* was found to have the highest number of stomata on the abaxial side of the leaf, while the other two species had a similar number (Table 2, Figure 5). The number of stomata on the adaxial side of the leaf (No_Stom_ad) has been shown to be the most plastic of the analysed traits in *G. dinarica*, in contrast to the other two species (Table 2, Figure 5). All other characters, length of the stomata on the adaxial side of the leaf (L_Stom_ad), length of the stomata on the abaxial side of the leaf (L_Stom_ab), width of the stomata on the adaxial side of the leaf (W_Stom_ad), and width of the stomata on the abaxial side of the leaf (W_Stom_ab), are similar among studied species and do not provide sufficient information for their delimitation (Table 2, Figure 5). A complete dataset with the results of the measurements for all characters and samples is provided in the Supplementary Material.

### 2.3. Coefficient of Variation

All characters except one showed a moderate degree of variability (CV = 20–50%). Character that showed a high degree of variability with a coefficient of variation (CV%) of more than 50% was the number of crystal cells (No_CC) (73.29%) (Table 1). When analysing the variability of the characteristics within the taxa, it was found that *G. acaulis* is the only species in which the number of crystal cells shows significant variability. In contrast, the other two species show moderate variability in all their characteristics (Table 2).

### 2.4. Kruskal–Wallis Test

A Kruskal–Wallis test has shown that almost all the characters exhibit statistically significant contributions (*p* < 0.05) to the differentiation of the analysed taxa (Table 2). The characters that have not shown any statistical significance in anatomical differentiation pertain to those associated with leaf epidermis: number of stomata on the adaxial side of the leaf (No_Stom_ad), length of the stomata on the adaxial side of the leaf (L_Stom_ad), width of the stomata on the adaxial side of the leaf (W_Stom_ad), length of the stomata on the abaxial side of the leaf (L_Stom_ab), and width of the stomata on the abaxial side of the leaf (W_Stom_ab).

### 2.5. Correlative Variability

The analysis of the correlation between the characters of leaf anatomy has shown that only two characters are statistically significantly correlated (coefficient of correlation > 0.9) (Table 3). Those two characters are thickness of mesophyll (T_Mes) and thickness between the central nerve and the leaf margin (T_LH).

The analysis of the correlation between the characters of epidermis has demonstrated that no statistically significant correlation exists between the studied characters.

### 2.6. Multivariate Statistics

**Figure 6:**
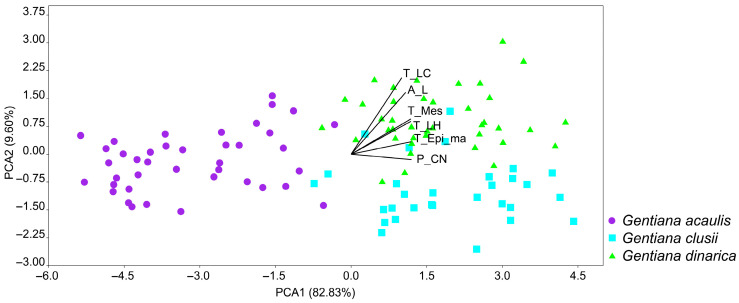
Biplot of principal component analysis (PCA) based on leaf anatomical characters of populations of the Balkan representatives of *Gentiana* section *Ciminalis*. Biplot shows the characters that contribute the most to the separation of the analysed populations along the first two axes: perimeter of the central nerve (P_CN), thickness between the central nerve and the leaf margin (T_LH), thickness of the mesophyll (T_Mes), thickness of the epidermis at the leaf margins (T_Epi_ma), thickness of the leaf in the area of the central nerve (T_LC), and surface area of the half leaf cross-section (multiplied by two in the statistical analysis) (A_L).

The principal component analysis (PCA) based on the analysed anatomical traits resulted in a relatively clear separation of the groups (Figure 6). The first two PCA axes explained 92.42% of the total variability, 82.83% and 9.60%, respectively. The results of the PCA, depicted in the PCA scatterplot, showed that *G. acaulis* is clearly separated from the other two species along the first PCA axis. In contrast, *G. dinarica* and *G. clusii* are completely overlapped along the first axis, but just slightly along the second PCA axis (Figure 6). The characters with the highest loadings contributing mainly to the first two axes are: perimeter of the central nerve (P_CN), thickness between the central nerve and the leaf margin (T_LH), thickness of the mesophyll (T_Mes), thickness of the epidermis at the leaf margins (T_Epi_ma) (first axis), thickness of the leaf in the area of the central nerve varies between (T_LC), and surface area of the half leaf cross-section (multiplied by two in the statistical analysis) (A_L) (second axis) (Table 1, Figure 6).

**Figure 7:**
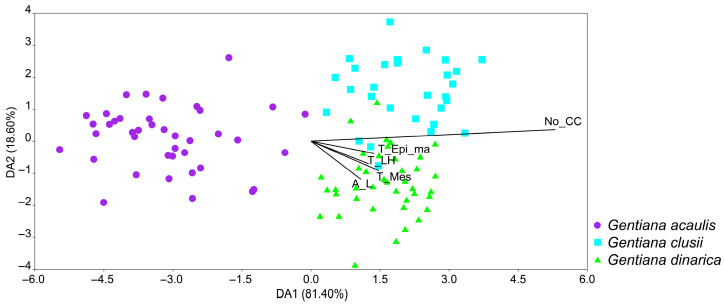
Biplot of discriminant analysis of the Balkan representatives of *Gentiana* section *Ciminalis* based on leaf anatomical characters. The three defined groups are three species–*Gentiana acaulis*, *G. clusii*, and *G. dinarica*. Biplot of the characters that contribute most to the separation of the species: number of crystal cells on the 1 mm-long portion of the leaf cross-section (No_CC), thickness of the mesophyll (T_Mes), thickness of the epidermis at the leaf margins (T_Epi_ma), thickness between the central nerve and the leaf margin (T_LH), and the surface area of the half leaf cross-section (multiplied by two in the statistical analysis) (A_L).

The discriminant analysis (LDA) based on the leaf anatomical characters showed a clear separation of *G. acaulis* from the other two species along the first discriminant axis, while *G. clusii* and *G. dinarica* are completely overlapped (Figure 7). Furthermore, the latter two species are separated almost completely along the second discriminant axis. The characters that contributed most to the separation of the species are: number of crystal cells on the 1 mm-long portion of the leaf cross-section (No_CC), thickness of the mesophyll (T_Mes), thickness of the epidermis at the leaf margins (T_Epi_ma), thickness between the central nerve and the leaf margin (T_LH), and the surface area of the half leaf cross-section (multiplied by two in the statistical analysis) (A_L). The classification function showed that the total percentage of correctly classified individuals was 93.64%. The percentage of correctly classified individuals for *G. acaulis* and *G. dinarica* was 95%, while the percentage for *G. clusii* was 90%.

**Figure 8:**
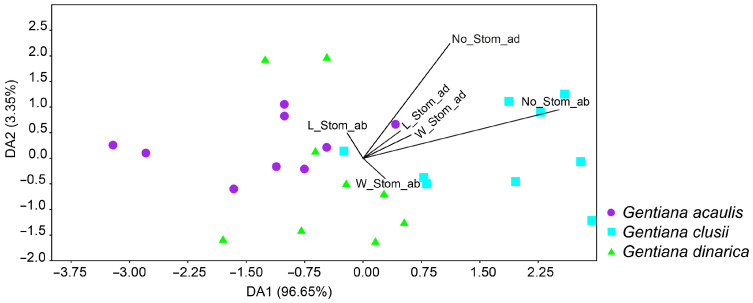
Biplot of discriminant analysis of the Balkan representatives of *Gentiana* section *Ciminalis* based on leaf epidermis anatomical characters. The three defined groups are three species—*Gentiana acaulis*, *G. clusii*, and *G. dinarica*. No_Stom_ad—number of stomata on the adaxial side of the leaf; No_Stom_ab—number of stomata on the abaxial side of the leaf; L_Stom_ad—length of the stomata on the adaxial side of the leaf; L_Stom_ab—length of the stomata on the abaxial side of the leaf; W_Stom_ad—width of the stomata on the adaxial side of the leaf; W_Stom_ab—width of the stomata on the abaxial side of the leaf.

The discriminant analysis (LDA) based on the leaf epidermis characters showed a separation of *G. clusii* from the other two species along the first discriminant axis, while *G. acaulis* and *G. dinarica* are completely overlapped (Figure 8). The character that contributed most to the separation of the species is number of stomata on the abaxial side of the leaf (No_Stom_ab).

## 3. Discussion

The complex interspecific relationships present within *Gentiana* section *Ciminalis*, in combination with the varying taxonomic interpretations documented in extant literature, have resulted in uncertainty regarding the taxonomic classification of some species within this section. Specifically, the taxonomic treatment of *G. acaulis*, *G. clusii*, and *G. dinarica* has been subject to variation, with some authors recognising them as distinct species [2,3,4,7,8,9], whereas others have classified *G. dinarica* as a subspecies of *G. acaulis* [14]. This study emphasises the significance of anatomical investigations in clarifying species boundaries within taxa that are otherwise challenging to classify, particularly in the case of *Gentiana* sect. *Ciminalis*.

A comparative analysis of the leaf anatomy of *Gentiana acaulis*, *G. dinarica*, and *G. clusii* revealed both common and species-specific characteristics. All species showed a dorsiventral laminar structure, but the degree of mesophyll differentiation varied between them. While *G. acaulis* showed a less-differentiated mesophyll structure dominated by isodiametric cells, *G. clusii* and *G. dinarica* showed a more distinct separation of palisade and spongy parenchyma, with *G. clusii* showing the most distinct stratification (Figure 3). This observation suggests a gradient in anatomical specialisation that could indicate ecological or phylogenetic divergence. As already mentioned, these three species live in mountainous regions of the temperate climate zone, which may explain the typical meso-morphic structure of the leaves. The difference in the stratification of the mesophyll can be explained by the different microclimatic conditions in the areas where each species live. *Gentiana acaulis* grows in acidic soils [4], which tend to remain cooler and wetter due to their compact structure and slower drainage [17]. In contrast, *G. clusii* and *G. dinarica* grow in dry, porous, soils developed on calcareous bedrock where soil moisture is volatile, favouring plants adapted to drought and high solar exposure [17]. These differences in water availability result in different ecological conditions, with species growing on siliceous bedrock being adapted to mesic or moderately moist environments, while xerophytic and termophilous taxa grow on calcareous bedrock [17]. This may explain the difference in mesophyll stratification between *G. acaulis* on the one side and *G. clusii* and *G. dinarica* on the other.

Quantitative anatomical traits such as leaf thickness (T_LC, T_LH), mesophyll thickness (T_Mes), and cuticle and epidermis dimensions varied significantly across species. The thickness of the mesophyll (T_Mes) has the greatest value in *G. dinarica* (mean value 365.85 μm), followed by *G. clusii* (mean value 305.35 μm), while the populations belonging to *G. acaulis* have the lowest values of mesophyll thickness (mean value 164.31 μm).

The descriptive statistics revealed moderate variability in most traits. The number of crystal cells (No_CC) exhibited the highest degree of variability (CV = 73.29%), thus indicating that this trait may be less reliable for consistent taxonomic separation or may be indicative of plasticity in response to environmental conditions, such as calcium availability. The populations belonging to *G. acaulis* have the lowest number of crystal cells, while the population from the Bjelasica Mountain has no crystal cells at all. The populations belonging to *G. clusii* have the largest number of crystal cells, while the populations of *G. dinarica* lie between these two species (Figure 3).

Calcium oxalate crystals have an acicular shape, are also found in several other *Gentiana* species [18,19,20], and are mostly located in the palisade layer. There are different opinions about the function of calcium oxalate crystals in plants, including maintenance of ionic equilibrium, removing of oxalate that can accumulate in toxic amounts, or that they merely serve as structural support or as a protective device against foraging animals [21,22]. Given that all three studied species inhabit stony grounds and grasslands, it can be assumed that one of the functions of the crystals is to protect against foraging animals. On the other hand, the findings of our research suggest a correlation between the presence or absence of these crystals and the type of substrate on which the studied species grow. The high concentration of calcium oxalate crystals observed in *G. clusii* and *G. dinarica* can be explained by the calcareous substrate on which they grow. In contrast, *G. acaulis*, which grows on siliceous substrate or on deep soils from which the lime has been removed by washing [4], exhibits a low concentration of these crystals. A significant aspect of our research pertains to the analysis of the presence of calcium oxalate crystals. These crystals were not identified in previous anatomical studies, including those conducted by [15,16].

A thorough examination of the epidermis of the leaf revealed that the majority of the analysed characters were not significant in distinguishing between the three species under investigation. The only character that proves significant in the separation of the examined species is the number of stomata on the abaxial side of the leaf (No_Stom_ab). The species *G. clusii* is distinguished by a greater number of stomata on the abaxial side of the leaf when compared to the other two species, which exhibit a lower yet comparable number of stomata on this specific side of the leaf. The greater number of abaxial stomata in *G. clusii* compared to *G. aculis* and *G. dinarica* can be explained by the different ecological conditions under which these species occur. *G. acaulis* is restricted to acidic, siliceous soils [4], which are compact and cooler and retain moisture better due to slower drainage [17], which reduces water loss across the adaxial surface. In contrast, *G. clusii* grows on porous, calcareous soils in high montane and subalpine grasslands and on rocky slopes [4], where water availability is highly variable and rapid drainage increases the risk of desiccation during intense solar radiation [17]. Under these dry conditions, the concentration of stomata on the abaxial side of the leaf reduces transpiration loss while maintaining efficient carbon assimilation. Although *G. dinarica* also grows on calcareous or dolomitic substrates, it is mostly restricted to steep rocky outcrops [4], where the buffering effects of rock surfaces and slopes can mitigate microclimatic extremes and reduce direct exposure [23] compared to the open grasslands typical of *G. clusii*. Thus, among the three species, *G. clusii* experiences the most water-limited and radiation-intense conditions, which favours a more conservative stomatal arrangement with higher abaxial density.

Multivariate analyses (PCA and LDA) supported the anatomical distinctiveness of *G. acaulis*, which was clearly separated from the other two species primarily along the first PCA and LDA axes. While *G. clusii* and *G. dinarica* exhibited considerable overlap in PCA and LDA space, their separation along the second axes indicates that fine-scale anatomical traits, such as thickness of the leaf in the area of the central nerve (T_LC) and the surface area of the half leaf cross-section (multiplied by two in the statistical analysis) (A_L), are effective for distinguishing them when used in combination.

Principal component analysis revealed that the traits that contributed most to variability and were described on the first two axes (92.42%) belong to the group of moderately variable characters (CV = 20–50%). Thickness of the mesophyll (T_Mes) and thickness of the leaf in the area of the central nerve (T_LC) are the characters that contribute most to the separation of populations.

In discriminant analysis, the characters that contributed most to the differentiation of the species studied were the number of crystal cells (No_CC) and the thickness of the mesophyll (T_Mes). Although the number of crystal cells exhibited the highest degree of variability, its high discriminatory power in the LDA suggests the potential for implementation in taxonomy when considered alongside other traits. The high classification success rate (93.64%) emphasises the reliability of the anatomical features of the leaves in the identification of species within this complex group of Gentians on the Balkan Peninsula.

In conclusion, the results presented here showed that *Gentiana acaulis*, *G. clusii*, and *G. dinarica* can be distinguished with a high degree of accuracy based on the anatomical features of the leaves. The anatomical variation observed among these species is indicative of both taxonomic boundaries and potential ecological adaptations. These findings highlight the value of detailed anatomical analysis in taxonomic studies and may serve as a foundation for further ecological or evolutionary investigations in the *Gentiana* genus. Moreover, further detailed morphological and genetic studies are required prior to the suggestion of a revised taxonomic treatment of *Gentiana* section *Ciminalis*.

## 4. Materials and Methods

**Figure 9:**
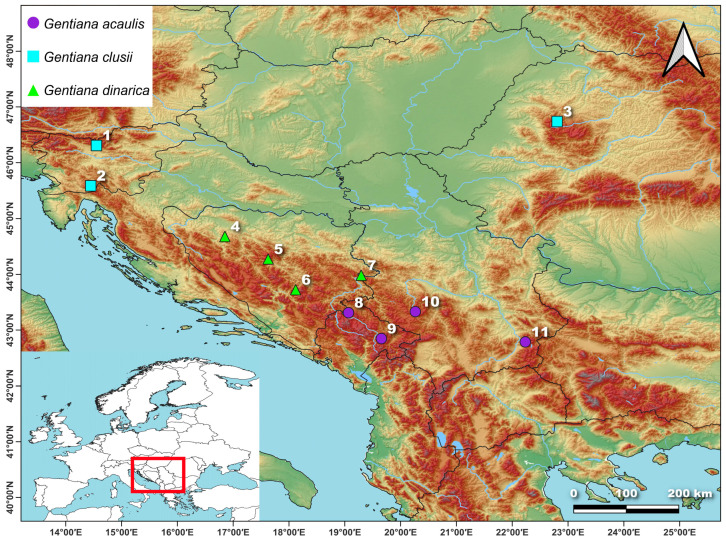
Sampled populations of the Balkan representatives of *Gentiana* section *Ciminalis*. Population identifiers correspond to Table 4.

A total of 110 individuals from 11 populations were included in the anatomical analyses. In order to avoid the influence of seasonal variation in leaf development or phenology, we collected all plants at the same phenophase, at the flowering stage, to ensure consistent sampling. The samples were collected from April to June 2024, from 10 populations in their natural habitats on the Balkan Peninsula, while one population originated from the Carpathian Mountains (Table 4, Figure 9). From each population, entire plants of 10 individuals were collected and fixed in the field in a mixture of glycerol and 50% ethanol (1:1). Between 1 and 5 specimens were selected for vouchers, which were deposited in the Herbarium of the Institute of Botany and Botanical Garden “Jevremovac”, Faculty of Biology, University of Belgrade (BEOU).

**Figure 10:**
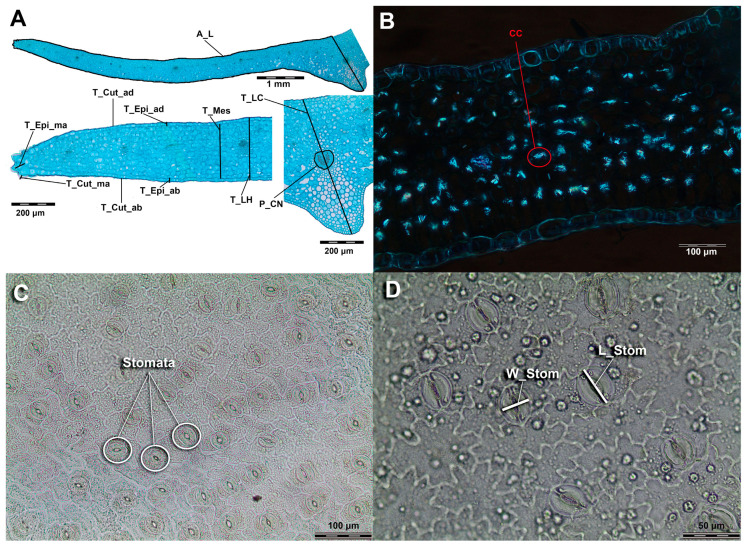
(**A**) Leaf cross-section obtained from an individual of *Gentiana dinarica* from Tara with measured characters: perimeter of the main vascular bundle (P_CN), thickness of the leaf in the area of the central nerve (T_LC), thickness between the central nerve and the leaf margin (T_LH), thickness of mesophyll (T_Mes), thickness of the epidermis on the adaxial side (T_Epi_ad), thickness of the epidermis on the abaxial side (T_Epi_ab), thickness of the epidermis at the leaf margins (T_Epi_ma), thickness of the cuticle on the adaxial side (T_Cut_ad), thickness of the cuticle on the abaxial side (T_Cut_ab), thickness of the cuticle at the leaf margins (T_Cut_ma), and surface area of the half leaf cross-section (multiplied by two in the statistical analysis) (A_L). (**B**) Leaf cross-section obtained from *Gentiana clusii* from Krvavec with counted character: crystal cell on the 1 mm-long portion of the leaf cross-section (CC). (**C**) Epidermis of the leaf, magnification 20×, obtained from an individual of *Gentiana clusii* from Krvavec. The number of the stomata were counted on the surface area of 0.324 mm^2^ (this character was measured on both adaxial and abaxial side of the leaf). (**D**) Epidermis of the leaf, magnification 40×, obtained from an individual of *Gentiana dinarica* from Hranisava with measured characters: width of the stomata (W_Stom) and length of the stomata (L_Stom) (these characters were measured on both adaxial and abaxial side of the leaf).

The leaf anatomy was examined on permanent slides, prepared by the standard method for light microscopy [24]. The 45 µm thick leaf cross-sections were prepared with a Reichert sliding microtome. The sections were cleared in Parazone and washed thoroughly in water. They were then stained with safranin (1% *w*/*v* in 50% ethanol) and alcian blue (1% *w*/*v*, aqueous). Epidermal peels were prepared using Jeffrey’s solution (10% nitric acid and 10% chromic acid, 1:1) (3 individuals per population; populations from Golija (GA_Gol) and from Vlašić (GD_Vla) have not been included in the analysis of leaf epidermis). After dehydration, the slides were mounted in Canada balsam [24]. The cross-sections of the leaves were imaged using an Olympus BX-41 trinocular microscope (Olympus Corporation, Tokyo, Japan) and an Olympus SC30 microscope camera (Olympus Corporation, Tokyo, Japan). For the observation of crystals, a polarising film was placed on the illumination tube and above the specimen [25]. Counting of stomata and crystal-containing cells, as well as measurements of anatomical characters, were performed using DIGIMIZER image analysis software (2005-2011 MedCalc Sofware) [26]. A total of 12 anatomical characters were measured (Figure 10).

Descriptive statistics (mean, standard deviation, minimum, maximum and standard error, coefficient of variation) were calculated for each character. A Kruskal–Wallis test was performed to identify significant differences in analysed characters between the studied species. Principal component analysis (PCA) was performed on the entire data set to show the general pattern of variation along the first two components. Before performing the principal component analysis, the data were standardised and the calculations were based on the covariance matrix. For the analysis, the three species (*G. acaulis*, *G. clusii*, and *G. dinarica*) recognised by several authors [2,3,4,7,8,9] are referred to here as “defined groups”. The hypothesis regarding anatomical differences in the leaf between the “defined groups” was evaluated using discriminant analysis (LDA). The classification function was used to determine the percentage of correctly classified individuals in each group. Statistical analyses were performed using the Past 4.17c package [27].

## Figures and Tables

**Table 1:** Descriptive and multivariate statistics of studied leaf anatomical characters. Valid N—number of measurements, Mean—mean value, Min.—minimal value, Max.—maximal value, Std. Dev.—standard deviations, CV%—coefficient of variation. Factor 1 and Factor 2—factor loadings obtained in PCA.

Full Character Name	Acronym	Valid N	Min.	Max.	Mean	Std. Dev.	CV%	Factor 1	Factor 2
Perimeter of the main vascular bundle (μm)	P_CN	110	39.36	112.74	67.05	15.40	22.97	0.31	−0.04
Thickness of the leaf in the area of the central nerve (μm)	T_LC	110	343.69	1334.38	641.66	155.08	24.17	0.26	0.52
Thickness between the central nerve and the leaf margin (μm)	T_LH	110	141.76	572.91	328.38	111.24	33.87	0.30	0.23
Thickness of the epidermis on the adaxial side (μm)	T_Epi_ad	110	13.98	36.56	21.71	5.14	23.67	0.28	−0.31
Thickness of the epidermis on the abaxial side (μm)	T_Epi_ab	110	13.42	32.17	21.18	4.66	22.00	0.29	−0.26
Thickness of the epidermis at the leaf margins (μm)	T_Epi_ma	110	17.83	102.79	52.18	18.65	35.75	0.30	0.09
Thickness of the cuticle on the adaxial side (μm)	T_Cut_ad	110	2.57	9.62	5.20	1.60	30.80	0.29	−0.35
Thickness of the cuticle on the abaxial side (μm)	T_Cut_ab	110	2.42	8.45	4.75	1.33	27.91	0.29	−0.33
Thickness of the cuticle at the leaf margins (μm)	T_Cut_ma	110	3.93	17.59	9.10	2.29	25.18	0.29	−0.16
Thickness of the mesophyll (μm)	T_Mes	110	102.55	504.57	276.06	107.94	39.10	0.30	0.24
Number of crystal cells on the 1 mm-long portion of the leaf cross-section	No_CC	110	0.00	210.00	63.29	46.39	73.29	0.28	0.42
Surface area of the leaf cross-section (mm^2^)	A_L	110	0.73	7.28	2.81	1.39	49.49	0.28	0.00
Number of stomata on the adaxial side of the leaf (A = 0.324 mm^2^)	No_Stom_ad	27	7	45	24.78	8.74	35.25		
Number of stomata on the abaxial side of the leaf (A = 0.324 mm^2^)	No_Stom_ab	27	16	58	33.55	11.08	33.05		
Length of the stomata on the adaxial side of the leaf (μm)	L_Stom_ad	27	28.5	43.1	35.44	3.69	10.43		
Width of the stomata on the adaxial side of the leaf (μm)	W_Stom ad	27	23.41	36.9	29.34	3.65	12.47		
Length of the stomata on the abaxial side of the leaf (μm)	L_Stom_ab	27	31.3	42.69	35.98	2.84	7.88		
Width of the stomata on the abaxial side of the leaf (μm)	W_Stom_ab	27	23.3	35.36	30.42	3.03	9.99		

**Table 2:** Comparative table with descriptive statistics of studied leaf anatomical characters for three analysed species. Min—minimal value, Max—maximal value, Mean—mean value, SD—standard deviation, CV%—coefficient of variation.

								Kruskal–Wallis	
	Taxon	*Gentiana acaulis* Min–Max (Mean ± SD)	CV%	*Gentiana clusii* Min–Max (Mean ± SD)	CV%	*Gentiana dinarica* Min–Max (Mean ± SD)	CV%	H	*p*
Character									
Perimeter of the main vascular bundle (μm) P_CN		39.36–81.86 (54.76 ± 1.57)	18.15	51.99–97.38 (74.61 ± 2.22)	16.29	51.84–112.74 (73.67 ± 2.29)	19.7	43.7800	3.114 × 10^−10^
Thickness of the leaf in the area of the central nerve (μm) T_LC		343.69–752.85 (538.72 ± 15.68)	18.41	475.61–831.04 (621.50 ± 17.44)	15.37	533.06–1334.38 (759.72 ± 25.05)	20.86	46.1700	9.421 × 10^−11^
Thickness between the central nerve and the leaf margin (μm) T_LH		141.76–306.90 (213.25 ± 7.63)	22.62	210.13–565.31 (362.82 ± 14.59)	22.03	316.79–572.91 (417.69 ± 10.97)	16.61	73.9800	8.601 × 10^−17^
Thickness of the epidermis on the adaxial side (μm) T_Epi_ad		13.98–28.90 (18.43 ± 0.50)	17.05	17.09–36.56 (25.73 ± 1.03)	21.91	16.02–34.15 (21.96 ± 0.65)	18.62	37.8400	6.059 × 10^9^
Thickness of the epidermis on the abaxial side (μm) T_Epi_ab		13.42–24.83 (17.63 ± 0.41)	14.71	15.16–30.45 (24.87 ± 0.75)	16.6	15.19–32.17 (21.95 ± 0.66)	18.94	45.26	1.485 × 10^−10^
Thickness of the epidermis at the leaf margins (μm) T_Epi_ma		17.83–47.16 (32.20 ± 1.18)	23.17	40.40–90.31 (62.23 ± 2.55)	22.45	43.30–102.79 (64.62 ± 1.77)	17.96	63.62	1.533 × 10^−14^
Thickness of the cuticle on the adaxial side (μm) T_Cut_ad		2.57–5.92 (3.81 ± 0.13)	20.77	3.41–9.62 (6.82 ± 0.26)	20.87	3.31–8.26 (5.37 ± 0.16)	18.69	62.89	2.202 × 10^−14^
Thickness of the cuticle on the abaxial side (μm) T_Cut_ab		2.42–4.93 (3.61 ± 0.09)	16.51	3.68–8.45 (6.00 ± 0.22)	20.16	3.22–7.93 (4.97 ± 0.15)	19.05	75.54	3.954 × 10^−17^
Thickness of the cuticle at the leaf margins (μm) T_Cut_ma		3.93–9.73 (7.18 ± 0.25)	22.44	6.75–17.59 (10.78 ± 0.39)	19.97	6.86–12.67 (9.75 ± 0.24)	15.36	72.32	1.976 × 10^−16^
Thickness of the mesophyll (μm) T_Mes		102.55–255.87 (164.31 ± 6.99)	26.9	170.93–504.25 (305.35 ± 14.03)	25.17	261.89–504.57 (365.85 ± 10.58)	18.3	50.45	1.107 × 10^−11^
Number of crystal cells on the 1 mm-long portion of the leaf cross-section No_CC		0–128 (19.33 ± 4.43)	144.94	36–210 (105.60 ± 7.06)	36.64	11–127 (75.53 ± 3.88)	32.51	72.19	2.106 × 10^−16^
Surface area of the leaf cross-section (multiplied by two in the statistical analysis) (mm^2^) A_L		0.74–2.89 (1.57 ± 0.08)	31.42	1.72–4.28 (2.77 ± 0.14)	27.26	1.21–7.28 (4.08 ± 0.19)	29.94	62.92	1.887 × 10^−14^
Number of stomata on the adaxial side of the leaf (A = 0.324 mm^2^) No_Stom_ad		14–45 (22.89 ± 8.88)	38.86	21–32 (27.32 ± 4.17)	15.3	7–42 (24.10 ± 11.82)	49.01	3.821	0.1457
Number of stomata on the abaxial side of the leaf (A = 0.324 mm^2^) No_Stom_ab		18–39 (26.32 ± 5.70)	21.65	31–58 (44.67 ± 9.89)	22.15	16–41 (29.67 ± 7.39)	24.93	13.82	0.000971
Length of the stomata on the adaxial side of the leaf L_Stom_ad		28.50–43.10 (34.31 ± 4.63)	13.47	34.84–42.61 (37.62 ± 2.87)	7.67	29.64–37.60 (34.36 ± 2.56)	7.47	4.49	0.1059
Width of the stomata on the adaxial side of the leaf W_Stom_ad		23.41–35.26 (27.96 ± 3.83)	13.73	26.80–36.90 (31.69 ± 3.24)	10.3	23.54–33.09 (28.38 ± 2.95)	10.43	4.596	0.1005
Length of the stomata on the abaxial side of the leaf L_Stom_ab		32.55–40.85 (36.95 ± 2.83)	7.64	31.30–38.65 (35.16 ± 2.53)	7.19	32.63–42.69 (35.82 ± 3.15)	8.83	2.201	0.3327
Width of the stomata on the abaxial side of the leaf W_Stom_ab		23.30–34.80 (29.53 ± 3.84)	13.03	28.75–34.75 (31.32 ± 2.18)	6.95	25.53–35.36 (30.40 ± 2.92)	9.63	0.9136	0.6333

**Table 3:** Correlations of analysed anatomical characters of *Gentiana* section *Ciminalis* (highly correlated characters with the coefficient of correlation > 0.9, printed in red font). Perimeter of the main vascular bundle (P_CN), thickness of the leaf in the area of the central nerve (T_LC), thickness between the central nerve and the leaf margin (T_LH), thickness of the epidermis on the adaxial side (T_Epi_ad), thickness of the epidermis on the abaxial side (T_Epi_ab), thickness of the cuticle on the adaxial side (T_Cut_ad), thickness of the cuticle on the abaxial side (T_Cut_ab), thickness of mesophyll (T_Mes), thickness of the epidermis at the leaf margins (T_Epi_ma), thickness of the cuticle at the leaf margins (T_Cut_ma), surface area of the half leaf cross-section (multiplied by two in the statistical analysis) (A_L), and number of crystal cells on the 1 mm-long portion of the leaf cross-section (No_CC).

	P_CN	T_LC	T_LH	T_Epi_ad	T_Epi_ab	T_Cut_ad	T_Cut_ab	T_Mes	T_Epi_ma	T_Cut_ma	A_L	No_CC
P_CN		0.69	0.67	0.50	0.42	0.52	0.53	0.66	0.49	0.48	0.67	0.52
T_LC	0.69		0.73	0.38	0.36	0.34	0.38	0.75	0.55	0.46	0.75	0.39
T_LH	0.67	0.73		0.57	0.60	0.61	0.64	0.99	0.72	0.59	0.84	0.62
T_Epi_ad	0.50	0.38	0.57		0.71	0.60	0.57	0.55	0.50	0.52	0.45	0.51
T_Epi_ab	0.42	0.36	0.60	0.71		0.68	0.54	0.57	0.50	0.47	0.51	0.54
T_Cut_ad	0.52	0.34	0.61	0.60	0.68		0.76	0.60	0.56	0.52	0.45	0.62
T_Cut_ab	0.53	0.38	0.64	0.57	0.54	0.76		0.63	0.62	0.59	0.46	0.61
T_Mes	0.66	0.75	0.99	0.55	0.57	0.60	0.63		0.72	0.59	0.84	0.62
T_Epi_ma	0.49	0.55	0.72	0.50	0.50	0.56	0.62	0.72		0.63	0.71	0.61
T_Cut_ma	0.48	0.46	0.59	0.52	0.47	0.52	0.59	0.59	0.63		0.47	0.57
A_L	0.67	0.75	0.84	0.45	0.51	0.45	0.46	0.84	0.71	0.47		0.52
No_CC	0.52	0.39	0.62	0.51	0.54	0.62	0.61	0.62	0.61	0.57	0.52	

**Table 4:** Data on sampled populations. Population identifiers (ID), population acronyms, locality—sampling locality, coordinates—latitude and longitude in WGS84, No. individuals—number of individuals used from anatomical measurements, collector—collector information, voucher no.—voucher number. Vouchers are deposited in the herbarium of the Institute of Botany, Faculty of Biology, University of Belgrade (BEOU).

Taxon	ID	Population Acronym	Locality	Coordinate	Substrate	No. Individuals	Collector	Voucher No.
*Gentiana clusii*	1	GC_Krv	Slovenia, Kamnik-Savinja Alps, Krvavec	46.309453 N, 14.550278 E	Limestone	10	Glasnović, P.	72590
	2	GC_LiK	Slovenia, Dinarides, Liburnian karst	45.589039 N, 14.447636 E	Limestone	10	Glasnović, P., Surina, B.	72591
	3	GC_Pal	Romania, Carpathians, Pietrele Albe	46.7408835 N, 22.804003 E	Limestone	10	Kuzmanović, N., Lakušić, D., Mladenović, Ž.	72592
*Gentiana dinarica*	4	GD_Man	Bosnia and Herzegovina, Manjača, Donja Kozica	44.6791654 N, 16.8513912 E	Dolomite	10	Kuzmanović, N., Lakušić, D., Milanović, Đ., Mladenović, Ž.	72593
	5	GD_Vla	Bosnia and Herzegovina, Vlašić, Paklarske stijene	44.2745826 N, 17.6267689 E	Limestone	10	Kuzmanović, N., Lakušić, D., Mladenović, Ž.	72596
	6	GD_Hra	Bosnia and Herzegovina, Hranisava, Čulica	43.7253991 N, 18.1191356 E	Limestone	10	Kuzmanović, N., Lakušić, D., Bogunić, F., Mladenović, Ž.	72594
	7	GD_Tar	Serbia, Tara, Drlije	43.984059 N, 19.2942991 E	Limestone	10	Vukojičić, S., Kuzmanović, N., Mladenović, Ž.	72595
*Gentiana acaulis*	8	GA_Lju	Bosnia and Herzegovina, Ljubišnja, Konjsko polje	43.312776 N, 19.0654004 E	Silicate	10	Kuzmanović, N., Lakušić, D., Mladenović, Ž.	72589
	9	GA_Bje	Montenegro, Bjelasica, Troglava	42.848374 N, 19.6552107 E	Silicate	10	Kuzmanović, N., Lakušić, D., Mladenović, Ž.	72586
	10	GA_Gol	Serbia, Golija, Jankov kamen	43.3322284 N, 20.2632792 E	Silicate	10	Kuzmanović, N., Mladenović, Ž.	72588
	11	GA_Cem	Serbia, Čemernik, Mlačište	42.7892901 N, 22.2336746 E	Silicate	10	Stojković, S., Jovanović, A., Mladenović, Ž.	72587

## Data Availability

Data will be available upon request from the corresponding author.

## Supplementary Materials

The following supporting information can be downloaded at: https://www.mdpi.com/article/10.3390/plants14192977/s1, Raw data.
